# Nomenclatural changes in Cicadellidae: Typhlocybinae and Delphacidae (Homoptera)

**DOI:** 10.3897/zookeys.277.4273

**Published:** 2013-03-15

**Authors:** Dmitry A. Dmitriev, Stuart H. McKamey

**Affiliations:** 1 Illinois Natural History Survey, Prairie Research Institute, University of Illinois at Urbana-Champaign, 1816 S. Oak st., Champaign, IL 61820, USA; 2 USDA/ARS Systematic Entomology Laboratory, c/o National Museum of Natural History, MRC-168, Smithsonian Institution, 10th St. & Constitution Ave., PO Box 37012, Washington, DC 20013-7012

**Keywords:** Typhlocybinae, Delphacidae, leafhopper, planthopper, systematics, homonym, synonym

## Abstract

New replacement names are proposed for seven species of the subfamily Typhlocybinae; one new synonym is recognized in the family Delphacidae. The following changes are proposed: *Empoasca (Empoasca) angustata* **nom.nov.** for *Empoasca angusta* Linnavuori & DeLong (not Dworakowska); *Empoasca (Empoasca) chilensis* **nom.nov.** for *Empoasca diversa* Linnavuori & DeLong (not Vilbaste); *Austroasca verdensis* **nom.nov.** for *Empoasca artemisiae* Lindberg (not Lethierry); *Kropka vidanoi* Dworakowska for *Erythroneura unipunctata* Dlabola (not Cerutti); *Zyginella vietnamica* **nom. nov.** for *Zyginella melichari* Dworakowska (not Kirkaldy); *Eupteryx (Eupteryx) dlabolai* **nom. nov.** for *Eupteryx octonotata* Dlabola (not Hardy); *Baaora ahmedi* **nom. nov.** for *Baaora spinosa* (Ahmed) (not Beamer); *Paradelphacodes insolitus* Dmitriev is synonymized with *Paradelphacodes gvosdevi* (Mitjaev), **syn. nov.**

## Introduction

Use of the 3i software package (Dmitriev 2006) helped to reveal several junior homonyms in the leafhopper subfamily Typhlocybinae, which need publication of replacement names. The nomenclatural changes proposed in this paper are necessary to bring the classification of the subfamily Typhlocybinae into compliance with the International Code of Zoological Nomenclature (ICZN 1999). In addition, a new synonym in the family Delphacidae is recognized.

## Nomenclatural changes and notes

### Family Cicadellidae

Subfamily Typhlocybinae

***Empoasca (Empoasca) angustata* nomen novum** for *Empoasca angusta* Linnavuori & DeLong

*Empoasca angusta* Linnavuori & DeLong, 1977:17, described from Chile, is a junior primary homonym of *Empoasca (Buhria) angusta* Dworakowska, 1976:26, described from Cameroon.

Etymology: The original species name in modified spelling is used.

***Empoasca (Empoasca) chilensis* nomen novum** for *Empoasca diversa* Linnavuori & DeLong

*Empoasca diversa* Linnavuori & DeLong, 1977:179, described from Chile, is a junior primary homonym of *Empoasca (Matsumurasca) diversa* Vilbaste, 1968:87, an eastern Palaearctic and Oriental species.

Etymology: The species is named for the country, from which it was originally described.

***Austroasca verdensis* nomen novum** for *Empoasca artemisiae* Lindberg

*Austroasca artemisiae* (Lindberg), Dworakowska 1970b: 712

*Empoasca artemisiae* Lindberg, 1958: 208, described from Cape Verde, is a junior primary homonym of *Empoasca artemisiae* Haupt, 1924:294 (= *Austroasca vittata* (Lethierry, 1884:65)), a wide spread Palaearctic and Oriental species.

Etymology: The species named for the country of the original description.

***Kropka vidanoi* Dworakowska, 1970a: 619** **nomen revived**

= *Erythroneura unipunctata* Dlabola, 1957:119

*Empoasca unipunctata* Dlabola, described from southern Europe, is a junior primary homonym of *Empoasca tithide* var. *unipunctata* Cerutti, 1939:85 (= *Zygina (Zygina) tithide* Ferrari, 1882:159), another European species. Dworakowska (1981) recognized *Kropka vidanoi* Dworakowska as junior synonym of *Kropka unipunctata* (Dlabola). We restore *Kropka vidanoi* Dworakowska as a valid name for the species.

***Zyginella vietnamica* nomen novum** for *Zyginella melichari* Dworakowska

*Zyginella melichari* Dworakowska, 1977:24, described from Vietnam, is a junior secondary homonym of *Zyginella melichari* (Kirkaldy, 1906:315, *Eupteryx*), a species known from Sri Lanka.

Etymology: The species is named for the country, from which it was originally described.

***Eupteryx (Eupteryx) dlabolai* nomen novum** for *Eupteryx (Eupteryx) octonotata* Dlabola

*Eupteryx octonotata* Dlabola, 1974:51, described from Iran, is a junior secondary homonym of *Eupteryx octonotata* (Hardy, 1850:419, *Typhlocyba*) (= *Eupteryx (Eupteryx) urticae* (Fabricius, 1803: 77)), a wide spread Palaearctic species.

Etymology: The species is named for the author of the replaced name.

***Baaora ahmedi* nomen novum** for *Typhlocyba spinosa* Ahmed

*Baaora spinosa* (Ahmed), Dworakowska, 1982:104

*Typhlocyba spinosa* Ahmed, 1971:195, described from Pakistan, is a junior primary homonym of *Empoa (Empoa) spinosa* (Beamer, 1943:131, *Typhlocyba*), a wide spread Nearctic species.

Etymology: The species is named for the author of the replaced name.

***Typhlocyba rubrasvulva* var. *bifasciata* Osborn, 1898:12** **nom.nud.**

The name is a primary homonym of *Zonocyba bifasciata* (Boheman, 1851:212) (Original genus *Typhlocyba*). However, the name does not need a replacement, since it is not available.

**Family Delphacidae**

***Paradelphacodes gvosdevi* (Mitjaev, 1980)**

*Chloriona gvosdevi* Mitjaev, 1980: 36

*Paradelphacodes gvosdevi* Mitjaev, 2002: 104

= Para*delphacodes insolitus* Dmitriev, 2000: 281 **syn.n.**

Dmitriev (2002) described *Paradelphacodes insolitus* from Southern Russia, not far from the type locality of the *Chloriona gvosdevi* Mitjaev in Northern Kazakhstan. In 2002, Mitjaev compared his material to the illustrations of Dmitriev (2000) and transferred *Chloriona gvosdevi* Mitjaev to the genus *Paradelphacodes* without formally synonymizing both species. In this paper the synonymy of the two species is confirmed.
